# Idiopathic toe walking and sensory processing dysfunction

**DOI:** 10.1186/1757-1146-3-16

**Published:** 2010-08-16

**Authors:** Cylie M Williams, Paul Tinley, Michael Curtin

**Affiliations:** 1 Southern Health- Cardinia Casey Community Health Service, Locked Bag 2500, Cranbourne, VIC, 3977, Australia; 2 Charles Sturt University, School of Community Health, PO Box 789, Albury, NSW 2640 Australia; 3 School of Community Health, Thurgoona Campus, PO Box 789, Thurgoona, NSW 2640. Australia

## Abstract

**Background:**

It is generally understood that toe walking involves the absence or limitation of heel strike in the contact phase of the gait cycle. Toe walking has been identified as a symptom of disease processes, trauma and/or neurogenic influences. When there is no obvious cause of the gait pattern, a diagnosis of idiopathic toe walking (ITW) is made. Although there has been limited research into the pathophysiology of ITW, there has been an increasing number of contemporary texts and practitioner debates proposing that this gait pattern is linked to a sensory processing dysfunction (SPD). The purpose of this paper is to examine the literature and provide a summary of what is known about the relationship between toe walking and SPD.

**Method:**

Forty-nine articles were reviewed, predominantly sourced from peer reviewed journals. Five contemporary texts were also reviewed. The literature styles consisted of author opinion pieces, letters to the editor, clinical trials, case studies, classification studies, poster/conference abstracts and narrative literature reviews. Literature was assessed and graded according to level of evidence.

**Results:**

Only one small prospective, descriptive study without control has been conducted in relation to idiopathic toe walking and sensory processing. A cross-sectional study into the prevalence of idiopathic toe walking proposed sensory processing as being a reason for the difference. A proposed link between ITW and sensory processing was found within four contemporary texts and one conference abstract.

**Conclusion:**

Based on the limited conclusive evidence available, the relationship between ITW and sensory processing has not been confirmed. Given the limited number and types of studies together with the growing body of anecdotal evidence it is proposed that further investigation of this relationship would be advantageous.

## Background

A typical presentation of idiopathic toe walking (ITW) is that of a child who can heel-toe walk on request but habitually walks on his or her forefoot. ITW was first noted in a case review in 1967 [1] and was diagnosed as a congenitally short tendo-achilles. This condition is no longer found in the literature; instead reference is made to a clinical condition called habitual, or more recently, idiopathic toe walking (ITW) [2-4]. ITW is a diagnosis that can only be made in the absence of any medical condition known to cause toe walking [2,3]. Table 1[5-24] shows a range of medical conditions associated with toe walking.

**Table 1:** Known conditions associated with or causing toe walking

Accidents causing fractures/soft tissue injuries [5]
Angelman syndrome [6]

Anklyosing spondlyitis [7]

Autistic spectrum disorders [8]

Cerebral Palsy [9,10]

Charcot-Marie-Tooth [11]/Hereditary motor sensory neuropathy type 1A [12]

Congenital talipes equinus [13]

Developmental Coordination Disorder (Previously known as Clumsy Child Syndrome) [13]

Global developmental delay [14]

Leg length discrepancy [15]

Muscular Dystrophy [16]

Puncture wounds [17]

Scarring from accidents or burns [18]

Schizophrenia [19]

Spina Bifida [20]

Tethered cord syndrome [21]

Transient focal dystonia of the lower leg muscles [22]

Tumour within the belly of the gastrocnemius muscle [23]

Venous malformation of the gastrocnemius muscle [24]

The incidence of ITW has been reported as present in up to 7% of the general paediatric population in one small study [25]. As this study had small participant numbers and large variances in cultural influences, it is suggested that the findings are not a true indication of the prevalence of ITW. The incidence of family members reporting an ITW history, according to a number of retrospective studies [2,26-29], is between 10-88% with the only prospective study reporting an incidence of 34.1% (95% CI) [3]. It is also noted that having family members who have displayed ITW did not conclusively lead to further generations presenting with this gait type. ITW presents in either gender [3,27], with no gender displaying predominance. A link between speech delay, with or without a gross motor skill delay, and ITW has been identified in a number of studies [2,30-32]. There is an absence of research into the habitual nature of ITW or the social or familial influences of this gait style and no literature on any influential factors contributing to the initial development of this gait pattern.

The majority of literature on ITW focuses on its relationship to equinus. The suggestion that equinus is secondary to the development of this gait abnormality is common, but as yet unproven [2]. The presence of ITW post orthopaedic treatment and beyond has now been highlighted both in case controlled studies [3,33,34] and within a case report [35] leading to conflicting opinions on the necessity and effectiveness of treatment. The long-term effect that ITW has on the foot and ankle has not been definitively established, although it has been noted that there is some evidence of excessive external tibial rotation present in long-term toe walkers [36] and a positive association with equinus [2,27,36]. There continues to be debate within the literature of the need for treatment based on these associations.

Studies on the treatment of ITW include: comparative outcome analysis of surgical achilles tendon lengthening compared to or in combination with serial casting [36-39]; anecdotal case reviews [1,40]; and retrospective studies [28,33,38] on many different conservative treatment regimes which primarily involve stretching and some form of orthotic therapy or footwear. Two long-term retrospective studies report ITW persisting in children regardless of treatment received [28,33].

A number of authors within published texts suggest that ITW has a positive relationship with sensory processing dysfunction [14,41,42], yet there is limited research establishing this relationship. The content of these texts are currently utilised as teaching material within tertiary educational settings, all have been published or subsequent editions published within the last twelve years. Sensory processing is a conceptual model that is utilised in occupational therapy practice [43]. It has been defined as "the neurological process that organises sensation from one's own body and from the environment and makes it possible to use the body effectively within the environment" [44]. While sensory processing is a complex theory, it can be explained in terms of the primary and secondary senses of the body. There are five primary senses that the body relies on for its engagement within the environment. Vision or sight, hearing, touch, smell and taste are primarily the sensors that provide feedback from the environment. Secondary senses, vestibular and proprioception, provide feedback on where the body is in space, its location in response to other joints and its direction of movement [43,45]. According to sensory processing theory, the integration of the vestibular, proprioception and tactile systems are the building blocks on which normal body movement relies. These building blocks then form the base that allows children to develop their motor skills and cognition. These sensory systems, by the integration of the sensory input from the environment, then guide and organise motor control of the body. The sensory feedback that is obtained during and after an activity allows the result to be challenged within the brain and modified to gain the required result [42,46-48]. Sensory processing dysfunction (SPD) is a diagnosis given when these processes are not working appropriately.

The purpose of this paper is to examine the literature and provide a summary of what is known about the relationship between toe walking and SPD.

## Method

The last comprehensive literature review regarding ITW was published in 1999 [49], with recent ITW publications focusing on identification techniques and the critical evaluation of treatment options and subsequent outcomes.

An online search was conducted to identify literature exploring the relationship of the sensory system to toe walking. The search strategy was not limited to English. Japanese, French and German articles were also identified and translated. An inclusion preference was given to publications within peer reviewed journals, however published texts were also included. The primary search terms of 'toe' and 'walk', were used with a combination of the following terms: 'idiopathic', 'habitual', 'sensory', 'tactile', 'proprioception' and 'vestibular'.

The electronic databases searched included:

1. MEDLINE (1950-March 2010)

2. CINAHL (1960-March 2010)

3. AMI (1968-March 2010)

4. EBSCOhost (Health) (- March 2010)

5. The Cochrane Library (Databases: - March 2010)

6. Pubmed (1950- March 2010

7. Current Contents Connect (1998 - March 2010)

Published texts and references within relevant articles were also searched and included within this review. The results of this search are shown within Table 2[50-71].

**Table 2:** Idiopathic Toe Walking Literature

Contemporary Texts	Letters to the Editor	Comparative studies with/without control	Retrospective studies	Case studies	Expert opinion	Classifications	Literature reviews
Dunn, 1999 [50] Ayres, 2005 [43] Northern Territory Government, 2001 [51] Lane, 2002 [52] Tax, 1985 [53] Kranowitz, 1998 [42]	Accardo, 1997 [32] Cunningham, 1997 [54] Buie, 1975 [55]	McMulkin et al, 2006 [36] Fox et al, 2006 [3] Furrer & Deonna, 1982 [26] Hemo et al, 2006[37] Conrad & Bleck, 1980 [56] Brunt et al, 2004 [57] Li et al, 2004 [58] Brouwer et al, 2000 [39] Eastwood et al, 1997 [59] Crenna et al, 2005 [60] Accardo et al, 1992 [61] Kalen et al, 1986 [31] Kelly et al, 1997 [62] Shulman et al, 1997 [30] Sobel et al, 1997 [27] Stricker & Angulo, 1998 [2] Bernhard & Merkenschlager, 2008 [52] Taussig & Delouvee, 2001 [63] Bernhard et al, 2005 [64]	Eastwood et al, 2000 [33] Kogan & Smith, 2001 [38] Stott et al, 2004 [34] Hirsch & Wagner, 2004 [28]	Pomarino et al, 2007 [35] Hall et al, 1967 [1] Levine, 1973 [65] Williams & James, 2007[66] Herbertz et al, 2006 [12]	Caselli et al, 1988 [13] Pomarino & Bernhard,et al, 2006 [67] Pomarino, 2003 [40]	Alvarez et al, 2007 [68] Hicks et al, 1988 [9] Westberry et al, 2008 [69] Armand et al, 2006 [4] Griffin et al, 1977 [29]	Babb & Carlson, 2008 [70] Eiff et al, 2006 [71] Sala et al, 1999 [49]

The literature relating to ITW and sensory processing was reviewed according to the guidelines for evidence review set out by the National Health and Medical Research Council (NHMRC) [72]. These guidelines set out the process of evaluating the relationship of a medical condition's aetiology and risk factors (Table 3). This relationship can only be explored when there has been a clear association between the factor and disease. The guidelines also propose that the most appropriate research to determine this relationship is systematic reviews and observational studies. Within these, prospective or cohort studies provide stronger evidence than case-control studies [72].

**Table 3:** Designations of levels of evidence as proposed by NHMRC.

Level	Aetiology
I	A systematic review of level II studies

II	A prospective cohort study

III-1	All or none^§§§^

III-2	A retrospective cohort study

III-3	A case-control study

IV	A cross-sectional study/case series

§§§ All or none of the people with the risk factor(s) experience the outcome. For example, no smallpox develops in the absence of the specific virus; and clear proof of the causal link has come from the disappearance of small pox after large-scale vaccination.

Modified from "Designations of levels of evidence* according to type of research question (including tablenotes)" [72,73]

## Results

Three studies (Table 4) focused on identifying a possible link between a toe walking gait pattern and sensory processing. Toe walking within these studies was described as either idiopathic or toe walking associated with a medical condition.

**Table 4:** Literature selected for review

Study Author	Title	Study Type	Level of Evidence
Shulman et al, 1997 [30]	Developmental implications of idiopathic toe walking	Prospective, descriptive study without control	II

Bernhard and Merkenschlager, 2008 [25]	Beeinflusst Barfußlaufen die Häufigkeit des idiopathischen Zehenspitzengangs? - Unterschiede zwischen deutschen und bangalischen Kindern (Does the incidence of idiopathic toe walking change with barefoot walking? Differences between German and Bangladeshi children	Cross-sectional study	IV

Montgomery and Gauger, 1978 [14]	Sensory Dysfunction in children that toe walk	Case series	IV

Shulman et al [30], conducted a prospective, descriptive study without control, which involved gross motor skill and sensory processing tests on children diagnosed with ITW. The aim of the study was to examine the relationship between children who were diagnosed with idiopathic toe walking and their overall development. Thirteen children between the age of 19 months and six years and nine months were recruited based on their diagnosis of ITW. They concluded that the 13 participating children had significant delays in multiple developmental areas. However, they only tested nine of the 13 children for SPD as the remaining four children were either non-compliant or too young or old for the sensory test used. Of the nine children who underwent sensory testing, no significant sensory processing abnormalities were noted. The major limitations within this study were the small sample size and method of assessment of sensory processing. The choice of test, the DeGangi Berk Test of Sensory Integration (TSI) is a screening tool for children between the age of 3-5 years and not a tool that is able to diagnose sensory processing dysfunction. It is a tool that may be utilised when considering if more comprehensive sensory processing testing is appropriate. The TSI focuses on identifying vestibular and proprioceptive difficulties; however, does not assess the child's response to tactile, visual, hearing or taste stimuli. Although the researchers report that no child was diagnosed with sensory processing dysfunction, the testing method did not provide a comprehensive picture of each child's sensory processing function. Hence, it is not a sufficiently sensitive testing tool to identify a relationship between ITW and SPD and this study was unable to link the conditions.

Bernhard et al [25] conducted a cross-sectional study of 366 children in Germany and 1012 children living in the slums of Bangladesh. The authors developed reference norms from healthy and typically developing children in the general German population. Participant height, ankle range, case histories and sociological data were obtained from examination and questionnaires. ITW participants within the study were described as having a median age of 62 months, 68% were male, had a decreased ankle range of available dorsiflexion and typically came from families described as less educated. There was no information given about how the ankle range of movement was collected or how the family education levels were collected. The researchers went on to study children in Bangladesh and when compared to the original data, found a greater prevalence of ITW amongst the German participants (5.2%, n = 19) compared with the Bangladeshi participants (1.1% n = 11). The researchers attributed the lower rate of toe walking in Bangladeshi children to a lack of footwear, proposing the absence of footwear increased sensory input to the feet therefore decreasing the need for toe walking to gain this input. There was no data or testing noted within the article to support this supposition. Unequal cohort sizes, lack of standardised testing and limited recruitment information are major limitations of this study. On this basis, it is proposed that a definitive relationship between sensory processing and ITW could not be demonstrated by this study.

Only one study, which looked specifically at toe walking in children with significant developmental delays, found a possible relationship between toe walking and SPD. While there was no relationship between ITW and SPD explored within this paper, these authors are commonly cited for establishing a link between the toe walking gait pattern and SPD. Montgomery and Gauger [14] studied 17 children with significant developmental delay between the ages of six and 16. They aimed to explore sensory processing and its relationship to the toe walking gait pattern. During testing it was observed that all participants displayed mixed responses to tactile stimulation and all participants walked heel-toe after vestibular input/stimulation for short period of time. The researchers suggested that vestibular dysfunction is the primary cause of toe walking and that tactile defensiveness possibly exacerbates this. They also stated that toe walking maintains the stance phase of gait, prolonging stimulation of joint receptors, and thereby increasing proprioceptive input. It is thought that this increase allows the participant to better feel and process where their feet are placed during the gait cycle. Although the results of this suggest that the toe walking gait pattern has a link to SPD, it is possible that the developmental delay with which these participants were diagnosed, may be a reason for the mixed responsiveness to tactile stimulation and the changes in gait after vestibular input.

## Discussion

In her descriptive text, Kranowitz [42], stated that sensory processing does have a relationship with ITW. She discussed that when observing and treating children with a sensory processing disorder, some children toe walked. However, Kranowitz did not explore this relationship any further. This text was based on the author's opinion and was aimed at educating parents on SPD and how it may affect children's behaviour. This opinion is shared between a number of authors who have written texts and chapters about SPD [41,43,52], however this is not backed up with evidence.

Anecdotal references within contemporary texts, conference abstracts and case reports of sensory seeking behaviours [41,43,51,52,65], tactile defensiveness [40,42,52], poor proprioceptive awareness [43] or vestibular dysfunction [13,40], or the difficulty in modulation of combinations of these factors [40,50] being the initial cause of ITW were also found. All of these studies are graded at a Level IV, or the lowest form of evidence of an influencing aetiology due to their study design.

There are no recent studies that have included sensory processing within the study design or in the causality of ITW. This literature review has identified an increased interest in the relationship between ITW and sensory processing. Research into this potential relationship may supplement or improve the varied treatment options that are currently utilised for children that ITW. It is proposed that further research is required to determine if there is a link between SPD and ITW.

## Conclusion

Toe walking may be either a symptom of a medical condition or a gait pattern, the cause of which continues to elude researchers.

There is a small body of literature studying children who ITW and a possible link with SPD. The three studies that are most cited or relevant to the search terms do not provide conclusive proof that there is a link between ITW and SPD. Clinical observation and author opinion of ITW and potential relationship with SPD has increasingly been reported. These reports were found within published texts, conference abstract and case reports.

In the absence of comprehensive and evidence based studies, it is proposed that there is no proven link between ITW and SPD. Early exploration of a sensory relationship with the toe walking gait pattern and subsequent anecdotal evidence highlights the need for this link to be further investigated.

## Competing interests

The authors declare that they have no competing interests.

## Authors' contributions

CMW, conducted the systematic review, interpreted the findings and drafted the manuscript. PT & MC reviewed the manuscript and provided academic support throughout. All authors read and approved the final manuscript.
